# Higher fibrinogen and neutrophil-to-lymphocyte ratio are associated with the early poor response to intravenous thrombolysis in acute ischemic stroke

**DOI:** 10.3389/fneur.2024.1291950

**Published:** 2024-02-22

**Authors:** Mingzhu Deng, Kangping Song, Yangping Tong, Sufen Chen, Wei Xu, Guohua He, Jue Hu, Hui Xiao, Changmin Wan, Zhen Wang, Fangyi Li

**Affiliations:** ^1^Department of Neurology, Brain Hospital of Hunan Province, The Second People's Hospital of Hunan Province, Changsha, Hunan, China; ^2^Department of Neurology, The Affiliated Changsha Central Hospital, Hengyang Medical School, University of South China, Changsha, Hunan, China

**Keywords:** acute ischemic stroke, intravenous thrombolysis, fibrinogen, neutrophil-to-lymphocyte ratio, response

## Abstract

**Background:**

Inflammation and platelet activation play pivotal roles in acute ischemic stroke (AIS) pathogenesis. Early response to thrombolysis is a vital indicator for the long-term prognosis of AIS. However, the correlation between fibrinogen or the neutrophil-to-lymphocyte ratio (NLR) and the early response to intravenous thrombolysis in patients with AIS remains unclear.

**Methods:**

AIS patients undergoing intravenous thrombolysis were enrolled between January 2018 and May 2023. Blood cell counts were sampled before thrombolysis. A good response was defined as a National Institutes of Health Stroke Scale (NIHSS) score decreased ≥4 or complete recovery 24 h after thrombolysis treatment. A poor response was defined as any increase in the NIHSS score or a decrease in the NIHSS score <4 at the 24 h after thrombolysis treatment compared with that at admission. Logistic regression analysis was performed to explore the relationship of the fibrinogen level and NLR with a poor thrombolysis response. Receiver operating characteristic (ROC) analysis was used to assess the ability of the fibrinogen level and NLR to discriminate poor responders.

**Results:**

Among 700 recruited patients, 268 (38.29%) were diagnosed with a good response, and 432 (61.71%) were diagnosed with a poor response to intravenous thrombolysis. A binary logistic regression model indicated that an elevated fibrinogen level (odds ratio [OR], 1.693; 95% confidence interval [CI] 1.325–2.122, *P* < 0.001) and NLR (OR, 1.253; 95% CI, 1.210–2.005, *P* = 0.001) were independent factors for a poor response. The area under the curve (AUC) values for the fibrinogen level, NLR and fibrinogen level combined with the NLR for a poor response were 0.708, 0.605, and 0.728, respectively.

**Conclusions:**

Our research indicates that the levels of fibrinogen and NLR at admission can be used as a prognostic factor to predict early poor response to intravenous thrombolysis.

## Introduction

Stroke is a common neurological disease that results in neuronal cell damage due to inadequate blood supply to the brain or a loss of vascular integrity (1, 2). Stroke kills more than 15 million people annually, making it the second most prevalent cause of death globally (3). The major type of stroke is acute ischemic stroke (AIS), which is caused by a sudden arterial blockage (4). A crucial part of the etiology of AIS is played by inflammation and platelet activation (5). Patients with AIS can benefit from intravenous thrombolysis with recombinant tissue plasminogen activator (r-tPA) (6, 7). One cornerstone treatment of AIS is prompt intravenous r-tPA administration in the early phase (≤4.5 h) (6, 8, 9). Nevertheless, r-tPA has numerous drawbacks, including a relatively low recanalization rate among patients with large vessel occlusion, a risk of cerebral bleeding, which is closely related to patients' prognosis. Therefore, it is significant to explore the risk factors and the measurable biomarkers of early poor response to intravenous thrombolysis in AIS patients.

Neuroinflammatory response plays an essential role in the pathophysiology of ischemic stroke (10). Fibrinogen levels and neutrophil-to-lymphocyte ratio (NLR) are two common biomarkers of inflammation (11, 12). A meta-analysis has shown that a high fibrinogen level is an independent predictor for stroke in individuals who appear to be healthy (13). In AIS, fibrinogen has been identified as a biomarker for ischemic lesions (14). Through thrombogenesis (15) and inflammation (16), hyperfibrinogenemia can increase susceptibility to negative outcomes. However, investigations regarding the predictive value of fibrinogen levels after AIS remain contradictory (17–19). The NLR is an easily accessible serum biomarker and may be utilized for assessing systemic inflammation (12). As a prognostic indicator for coronary artery disease, peripheral arterial occlusive disease, and ischemic stroke, an increase in the NLR has been linked to atherosclerotic events (20–22). In particular, it has been found that an elevated NLR in stroke patients was associated with stroke severity, poor functional outcomes, and recurrent ischemic episodes (22, 23). Nevertheless, there is a relative paucity of studies on the correlation between the NLR and the early response to thrombolysis in patients with AIS.

Previous studies have confirmed that post-thrombolysis early neurological outcomes are relevant to the prognosis of patients treated with intravenous thrombolysis (24, 25). It is important to explore the risk factors of early poor response to intravenous thrombolysis in AIS patients. The correlation between fibrinogen or NLR and the early response to intravenous thrombolysis in patients with AIS remains unclear. In this study, we aimed to investigate whether fibrinogen and NLR can serve as predictive indicators for early response to r-tPA in patients with AIS.

## Materials and methods

### Study design and participants

**Figure 1:**
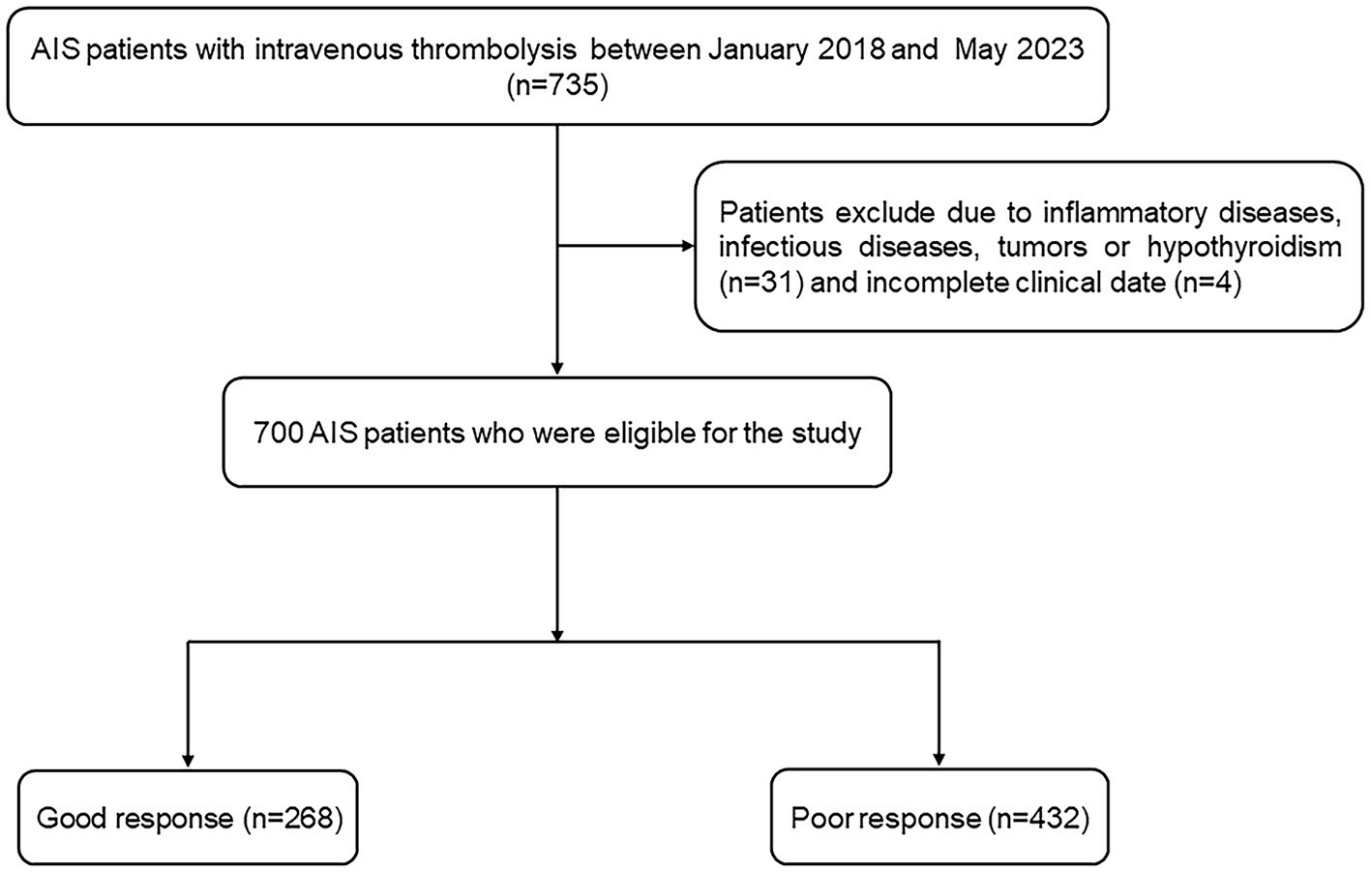
The study flow diagram. AIS, acute ischemic stroke.

AIS patients who underwent intravenous thrombolysis within 4.5 h were recruited from the Affiliated Changsha Central Hospital, Hengyang Medical School, University of South China. AIS patients were diagnosed with head imaging including CT and MRI, and the diagnostic criteria for AIS is based on Chinese guidelines for diagnosis and treatment of acute ischemic stroke 2018 (26). The indications and contraindications for intravenous thrombolysis are also based on Chinese guidelines for diagnosis and treatment of acute ischemic stroke 2018 (26). Eligible participants were enrolled in the final analysis if they met the following criteria: (1) admission within 4.5 h after onset; (2) treatment with intravenous thrombolysis with r-tPA; and (3) age 18 years or older. The exclusion criteria included: (1) the presence of severe inflammatory diseases or infectious diseases; (2) had immune system diseases, tumors or hypothyroidism; (3) had complications, such as severe liver and kidney injuries; and (4) incomplete clinical data. Informed consent was obtained from participants or their legal representatives. This study was approved by the Ethics Committee of the Affiliated Changsha Central Hospital, Hengyang Medical School, University of South China. There were 700 AIS patients recruited between January 2018 and May 2023, and a detailed flow diagram for patient enrollment is displayed in Figure 1.

### Data collection

Clinical assessments were performed in a blinded fashion by experienced neurologists. All participants underwent accurate and detailed records of their age, sex, body mass index, risk factors for stroke (hypertension, diabetes mellitus, atrial fibrillation, coronary artery disease, current drinking and smoking) on the day of admission. The lesion site and stroke subtype were determined by computed tomography or magnetic resonance. Stroke subtype was determined with the help of electrocardiography, echocardiography, carotid ultrasonography, and transcranial Doppler. Stroke subtype was classified according to the Trial of Org 10172 in Acute Stroke Treatment criteria (27). The demographic data, baseline clinical parameters, clinical diagnoses, and therapeutic schedules, were carefully extracted using a standardized case-report form. If any information was unclear, the doctors or other healthcare providers who had been in charge were consulted.

Blood samples of all patients were collected at 6–7 a.m the day after fasting for at least 8 h. Two milliliters of EDTA-anticoagulated whole blood were used for routine blood tests (automated hematology analyzer, BZ6800, China). Five milliliters of coagulant-containing blood was used for standard biochemical examination (automatic analyzer, HITACHI 7600, Japan). All the indicators were tested using commercial kits, which were operated by qualified professionals in accordance with the specifications. The counts of white blood cells (WBC), neutrophils, lymphocytes, monocytes, and platelets, as well as the levels of red blood cells (RBC), hemoglobin (Hb), and fibrinogen, were assessed in blood samples. The NLR, PLR, and LMR were calculated. The testing of blood was repeated three times for each.

### Definition of a good response and poor response to intravenous thrombolysis

Patients were classified into two groups, namely, the good response and poor response to intravenous thrombolysis groups. The good response was defined as a National Institutes of Health Stroke Scale (NIHSS) score decreased ≥4 or complete recovery 24 h after thrombolysis treatment. The poor response was defined as any increase in the NIHSS score or a decrease in the NIHSS score <4 at the 24 h after thrombolysis treatment compared with that at admission.

### Statistical analysis

Data analysis was performed by using SPSS 25.0 (IBM SPSS Statistics software, Version 25.0). All the data were tested for a normal distribution using the Kolmogorov–Smirnov test. Continuous variables were presented as mean ± SD, if they were normally distributed; otherwise, they were presented as median (quartile). The results for categorical variables are presented as percentages. Difference in baseline characteristics between groups were analyzed using Student's *t* test or Mann–Whitney U test for continuous variables as well as the chi-squared test or Fisher's exact test for categorical variables, as appropriate. We used box plots to show the distribution of the NLR, neutrophil count, lymphocyte count and fibrinogen level among the good response group and poor response group. Logistic regression analysis was used to detect risk factors for a poor response. A MedCalc 15.6.0 (MedCalc Software Acacialaan 22, B-8400 Ostend, Belgium) packet program was used to obtain a receiver operating characteristic (ROC) curve to test the overall ability of the NLR and fibrinogen level to discriminate poor responders to thrombolysis. A two-tailed value of *P* < 0.05 was considered significant.

## Results

### Clinical and demographic characteristics of patients with a good response and those with a poor response

**Figure 2:**
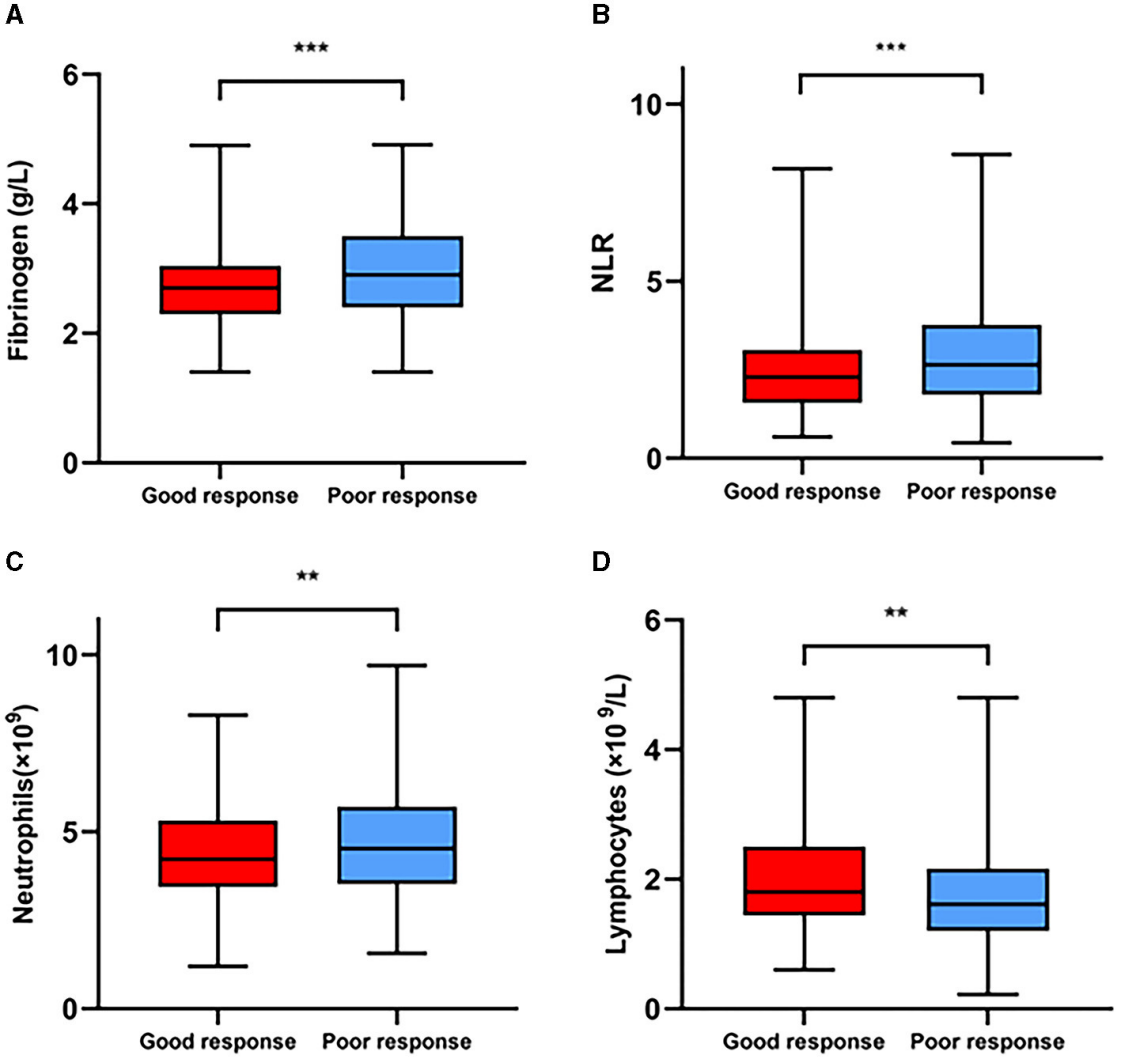
Comparisons of the plasma fibrinogen level **(A)**, NLR **(B)**, neutrophil count **(C)**, and lymphocyte count **(D)** between the good response and poor response groups. **P < 0.01, ***P < 0.001.

The demographic and clinical characteristics are comprehensively displayed in Table 1. A good response to thrombolysis was observed in 268 patients (38.29%), and a poor response was observed in 432 patients (61.71%). In poor response group, the age (*P* = 0.007), NIHSS score after rt-PA 24 h (*P* < 0.001), hypertension (*P* = 0.032), diabetes mellitus (*P* = 0.044), atrial fibrillation (*P* = 0.013), neutrophil count (*P* = 0.002), fibrinogen level (*P* < 0.001), NLR (*P* < 0.001), and PLR (*P* < 0.001) were significantly higher than those in good response group, while the lymphocyte count (*P* = 0.003) and Hb (*P* = 0.004) were significantly lower than those in good response group. In addition, the proportion of patients with symptomatic intracranial hemorrhage (sICH) was 5.79% for poor response group. Figure 2 shows the box plots of the fibrinogen level, NLR, neutrophil count and lymphocyte count for the two groups. The patients in the poor response group had an elevated fibrinogen level, NLR and neutrophil count compared with those in the good response group; however, the patients in the poor response group had lower lymphocyte counts (*P* < 0.05).

**Table 1:** Characteristics of patients with a good response and poor response to thrombolysis.

**Variable**	**Good response (268)**	**Poor response** **(432)**	**T/Z**	***P***
**Demographic characteristics**				
Age, years	64.65 ± 12.96	67.35 ± 12.62	−2.724	0.007
Male, *n* (%)	171 (63.80)	257 (59.49)	1.296	0.255
BMI, kg/m^2^	23.60 ± 3.22	23.36 ± 3.60	0.745	0.457
**Clinical assessment**				
NIHSS, score at admission	7 (3–10)	8 (4–11)	−1.453	0.146
NIHSS, score after rt-PA 24h	2 (1–4)	8 (4–15)	−14.648	< 0.001
sICH, *n* (%)		25 (5.79%)		
**Vascular risk factors**, ***n*** **(%)**				
Hypertension	181 (67.53)	324 (75.00)	4.583	0.032
Diabetes mellitus	54 (20.15)	116 (26.85)	4.041	0.044
Atrial fibrillation	35 (13.06)	88 (20.37)	6.103	0.013
Coronary artery disease	72 (26.87)	117 (27.08)	0.004	0.950
Current smoking	104 (38.80)	177 (40.97)	0.323	0.570
Current drinking	89 (33.21)	147 (34.02)	0.050	0.824
l**Stroke subtype**, ***n*** **(%)**			2.284	0.684
LAA	76 (28.36)	138 (31.94)		
SAO	133 (49.63)	198 (45.83)		
CE	47 (17.54)	75 (17.36)		
SOE	3 (1.12)	9 (2.08)		
SUE	9 (3.36)	12 (2.78)		
**Laboratory data**				
WBC (× 10^9^/L)	7.02 ± 1.82	7.22 ± 1.86	−1.340	0.181
Hb (g/L)	137.22 ± 18.88	133.17 ± 18.22	2.808	0.004
Neutrophils (× 10^9^/L)	4.44 ± 1.55	4.86 ± 1.86	−3.050	0.002
Lymphocytes (× 10^9^/L)	1.99 ± 0.75	1.78 ± 0.94	2.981	0.003
Platelets (× 10^12^/L)	212.89 ± 63.93	211.06 ± 63.56	0.366	0.714
Monocytes (× 10^9^/L)	0.41 ± 0.13	0.44 ± 0.53	−0.770	0.441
NLR	2.58 ± 1.47	3.65 ± 3.78	−4.389	< 0.001
PLR	122.37 ± 61.55	142.90 ± 81.13	−3.546	< 0.001
LMR	5.08 ± 1.96	4.70 ± 3.06	1.804	0.072
Fibrinogen (g/L)	2.75 ± 0.69	3.14 ± 0.94	−5.882	< 0.001

BMI, body mass index; sICH, symptomatic intracranial hemorrhage; LAA, large-artery atherosclerosis; SAO, small-artery occlusion; CE, cardioembolism; SOE, stroke of other determined etiology; SUE, stroke of undetermined etiology; WBC, white blood cell; Hb, hemoglobin; NLR, neutrophil-to-lymphocyte ratio; PLR, platelet-to-lymphocyte ratio; LMR, lymphocyte-to-monocyte ratio. Values are shown as number (percentage) or as medians (IQR) and mean (SD). Differences in baseline characteristics between groups were analyzed using Student's *t* test or Mann–Whitney U test for continuous variables as well as the chi-squared test or Fisher's exact test for categorical variables. A two-tailed value of *P* < 0.05 was considered significant.

### Logistic regression analysis for risk factors for a poor response

**Figure 3:**
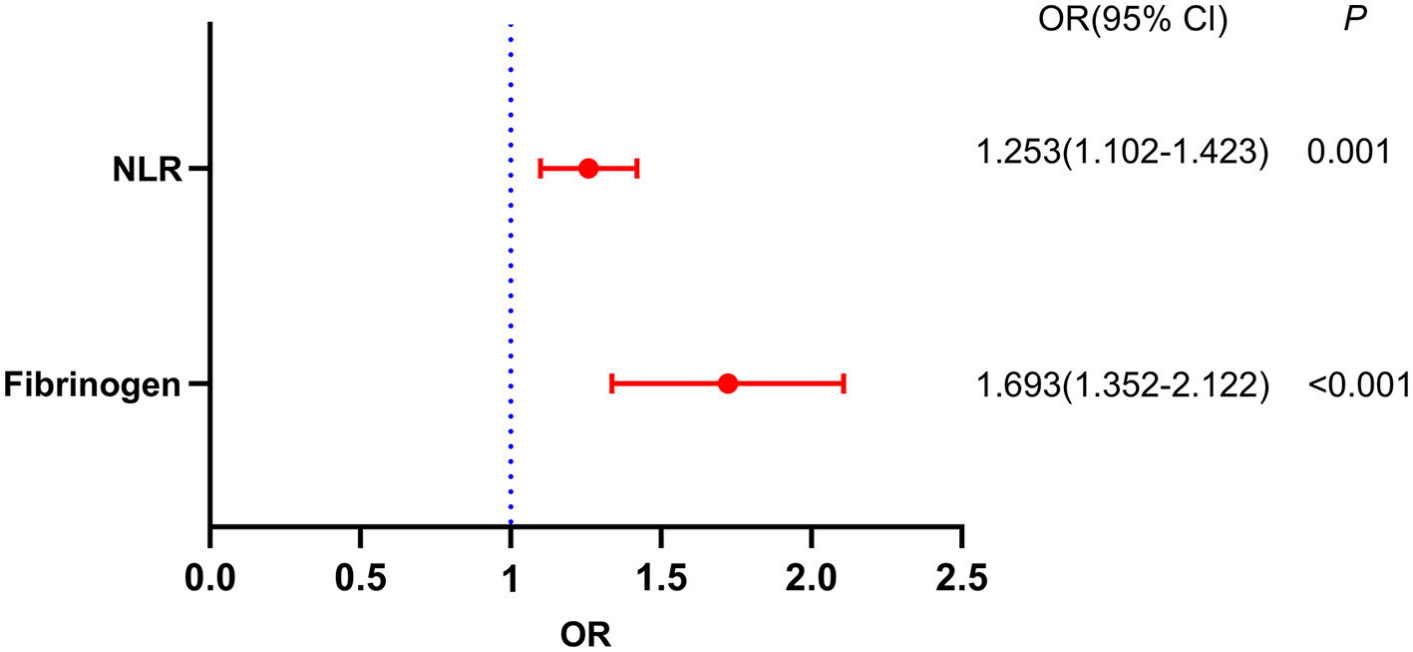
Binary logistic analysis of independent risk factors associated with a poor response to thrombolysis.

Table 2 illustrates the results of crude models for a poor response to thrombolysis. Factors with statistical significance in Table 1 were included in the binary logistic regression model to identify independent risk factors for a poor response to thrombolysis. Notably, the neutrophil and lymphocyte counts were not included in the model due to collinearity with the NLR and PLR. After adjustment for all potential confounders, such as age, hypertension, diabetes mellitus, atrial fibrillation, hemoglobin and PLR, an elevated NLR (OR, 1.253; 95% CI, 1.102–1.423, *P* = 0.001) and fibrinogen level (OR, 1.693; 95% CI, 1.352–2.122, *P* < 0.001) were identified as independent factors for a poor response to thrombolysis (Figure 3).

**Table 2:** Logistic regression analysis for risk factors for a poor response to thrombolysis.

**Variable**	**OR (95% CI)**	***P***	**Adjusted OR (95% CI)**	***P***
Age	1.017 (1.005–1.029)	0.007	1.006 (0.992–1.021)	0.376
Hypertension	1.442 (1.031–2.018)	0.033	1.329 (0.918–1.923)	0.293
Diabetes mellitus	1.455 (1.008–2.099)	0.045	1.165 (0.786–1.725)	0.447
Atrial fibrillation	1.703 (1.113–2.606)	0.014	1.501 (0.934–2.412)	0.094
Hb	0.988 (0.980–0.996)	0.005	0.995 (0.985–1.004)	0.276
Neutrophils	1.159 (1.052–1.277)	0.003		
Lymphocytes	0.756 (0.624–0.916)	0.004		
NLR	1.255 (1.142–1.378)	< 0.001	1.253 (1.102–1.423)	0.001
PLR	1.004 (1.002–1.007)	< 0.001	0.998 (0.994–1.002)	0.293
Fibrinogen	1.835 (1.483–2.271)	< 0.001	1.693 (1.352–2.122)	< 0.001

### ROC curve analysis to determine the overall ability to discriminate poor responders to thrombolysis

**Figure 4:**
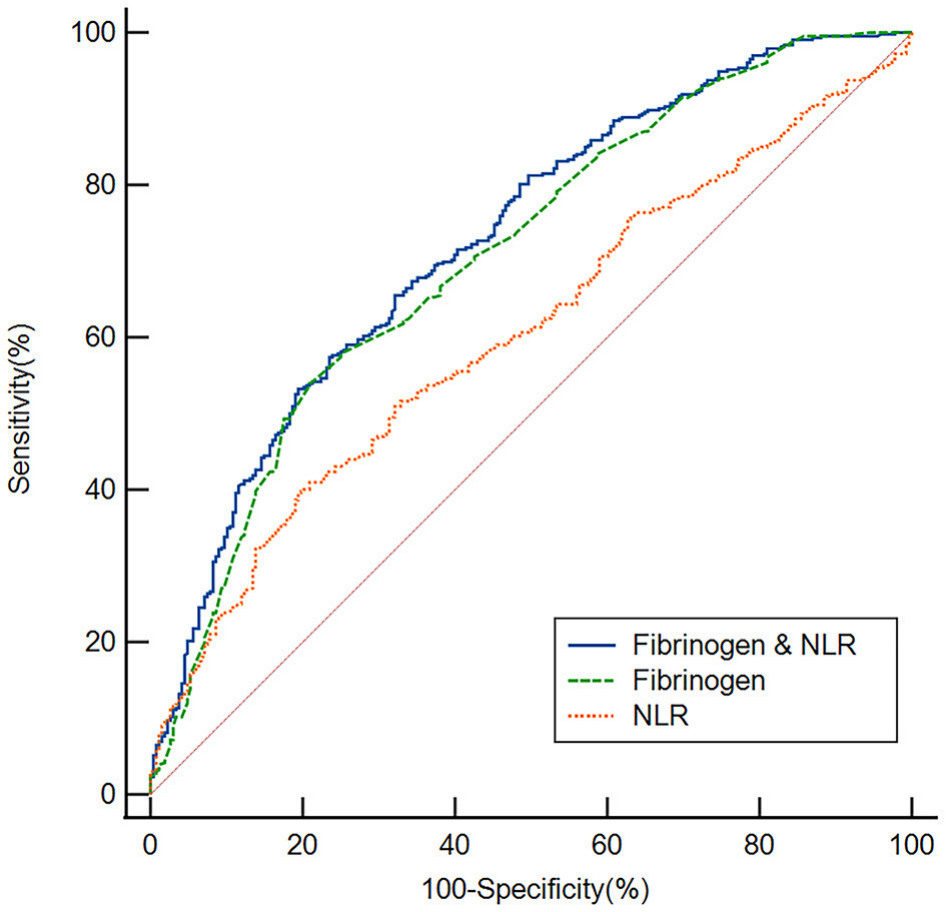
Based on ROC curve analysis, the fibrinogen level, NLR and fibrinogen level & NLR exhibited a respectable power to discriminate responders to thrombolysis, with AUC values of 0.708, 0.605 and 0.728, respectively.

We employed ROC curves to test the overall ability of the fibrinogen level and NLR to discriminate poor responders to thrombolysis (Figure 4). We observed that the area under the curve (AUC) of the fibrinogen level to discriminate poor responders to thrombolysis was 0.708 (95% CI, 0.673–0.742, *P* < 0.001), the optimal cutoff was 3.04, and the sensitivity and specificity were 53.94% and 79.10%, respectively. The AUC of the NLR was 0.605 (95% CI, 0.568–0.642, *P* < 0.001), the cutoff was 3.25, and the sensitivity and specificity were 39.58% and 80.60%, respectively. In addition, we conducted an ROC curve analysis for the ability of the combination of the fibrinogen level and NLR to discriminate between good responders and poor responders to thrombolysis. The AUC for the combination of the NLR and fibrinogen level was 0.728 (95% CI, 0.693–0.760, *P* < 0.001), the cutoff was 0.64, and the sensitivity and specificity were 57.41% and 76.49%, respectively.

## Discussion

Intravenous r-tPA has been the mainstay therapy for AIS for over 20 years (28). However, findings suggest that although some individuals do not respond to r-tPA, others experience a worsening of their clinical symptoms (such as symptomatic intracranial bleeding) (29, 30). Several studies have examined the long-term prognosis of AIS following intravenous thrombolysis or biomarkers to predict intracranial bleeding, while few studies have examined the early response to r-tPA (31, 32). In our research, a good response to thrombolysis was observed in 268 patients (38.29%), the ratio is also similar to the results of the previous studies (33, 34). Our findings provide several novel observations. First, we note that the plasma fibrinogen level and NLR in AIS patients with a poor thrombolysis response were higher than those in AIS patients with a good thrombolysis response. Second, the binary logistic regression model indicated that an elevated fibrinogen level and NLR were independent factors for a poor response. Finally, this study created fibrinogen level- and NLR-related ROC curves to distinguish AIS patients with a poor response from those with a good response to thrombolysis. Together, these data support that the fibrinogen level and NLR are associated with the response to thrombolysis.

Neuroinflammation has received increasing attention in recent years, and multiple studies have demonstrated that inflammatory processes are essential for the pathophysiology and development of ischemic stroke (35, 36). Fibrinogen can increase during any inflammatory event, where it serves to control systemic inflammatory signals (37, 38), and it is a major driver of blood viscosity involved in primary hemostasis, platelet aggregation, and interactions between leukocytes and endothelial cells (39). According to one study, fibrinogen might accumulate in inflammatory foci, and extravascular deposits worsen inflammation (40). Our study showed that fibrinogen levels were significantly higher in the poor response group than in the good response group. In addition, after adjustment for potential confounders, an elevated fibrinogen level was identified as an independent factor for a poor response to thrombolysis. Prior research has demonstrated an association between baseline elevated fibrinogen levels and an increased probability of short-term poor functional outcomes in AIS patients (41). Additionally, some research revealed that hyperfibrinogenemia was related to a poor functional result (42, 43). These results suggest that an elevated fibrinogen level is associated with a poor early response to thrombolysis in patients with AIS. Interestingly, as a defibrinogenating agent, the use of batroxobin alone decreased fibrinogen concentrations and erythrocyte aggregability, reduced stroke recurrence rates, and produced more significant improvements in neurological assessments (44). Thus, we speculate that drugs targeting fibrinogen may be valuable in improving the prognosis of AIS among specific patients. However, other studies showed no connection between fibrinogen levels and outcome (19, 45). We believe that the variances in the ethnicity of the research populations, the small sample sizes, the medication status, and the severity of the condition may be the causes of the discrepancies between various studies.

Neutrophils accumulate in the ischemic region during the early stages of stroke and produce inflammatory mediators, which disrupt the blood–brain barrier and cause an increase in the infarct volume, hemorrhagic transformation, and poor neurological outcomes (46, 47). Reducing the inflammatory response of peripheral neutrophils can improve regional cerebral blood flow and improve short-term and long-term functional outcomes after stroke (48). The production of matrix metalloproteinase-9 by peripheral neutrophils and monocytes might result in hemorrhagic transformation and a worsening of symptoms (49, 50). In contrast, lymphocytes, which are the brain's main regulators, can aid in both the healing of inflammatory damage and the restoration of brain function (51). In the present study, neutrophil counts were significantly higher in the thrombolytic poor response group than in the thrombolytic good response group; conversely, lymphocyte levels were decreased, which suggests the existence of peripheral inflammatory immune dysregulation in AIS. The NLR could serve as a peripheral indicator of the level of systemic inflammation (52), as this ratio incorporates information from both leukocyte subsets and supplementary immunological pathways. The NLR was shown to be able to predict the clinical prognosis in AIS patients in earlier research (34, 53, 54). It can predict hemorrhagic transformation following an ischemic stroke (55). Furthermore, studies have shown that an elevated NLR may indicate pneumonia associated with stroke (56). Our study reports a significantly higher NLR in the thrombolytic poor response group patients with AIS than in the thrombolytic good response group. Finally, after adjustment for potential confounders, an elevated NLR was identified as an independent factor for a poor response to thrombolysis. The results of our study have expanded on the role of the NLR in cerebrovascular illness and offer new viewpoints on therapeutic treatment.

In this study, we also discovered that age, hypertension, diabetes mellitus, atrial fibrillation, hemoglobin, and the PLR were related to a poor response to thrombolysis. Age and high baseline blood glucose levels were found to be risk factors for neurological recovery in earlier studies (49, 57, 58). AIS may lead to aberrant platelet function, and excessive platelet activation and buildup may impede stroke recovery (59). The PLR was shown to be able to predict the clinical prognosis in AIS patients in earlier research (60). Furthermore, the proportion of patients with sICH was 3.57% (25/700) for all AIS patients undergoing intravenous thrombolysis with r-tPA, which is consistent with a previous study (61).

According to estimates, AIS kills 1.9 million neurons every minute, and the concept that time is the brain is deeply rooted among the people. The earlier intervention may bring better outcome. Therefore, it is significant to explore the risk factors and the measurable biomarkers of early poor response to intravenous thrombolysis in AIS patients, it may help to save the neurological function of patient with AIS. The results of our ROC curve analysis showed that the fibrinogen level and NLR had appropriate sensitivity and specificity in distinguishing patients in the good response group from those in the poor response group. Clearly, the fibrinogen level is more discriminating than the NLR, suggesting that the fibrinogen level at admission may be a useful tool to predict a poor response to thrombolysis. Finally, we found that the combination of the fibrinogen level and NLR had a better ability to discriminate poor responders to thrombolysis, with an AUC of 0.728, which was higher than that of the individual markers, suggesting the use of markers in combination to predict a poor response to thrombolysis.

This study has the following limitations: (1) this is a retrospective study, and there may be potential inherent biases (such as selection bias); (2) this was a cross-sectional study, and all the participants enrolled were Chinese patients treated with intravenous thrombolysis, so our results need to be tested in non-Chinese populations, and longitudinal cohort studies with larger populations are required in the future; (3) there is a lack of other inflammatory markers to further assess whether the fibrinogen level and NLR are correlated with other inflammatory markers, which may hinder us from obtaining a comprehensive understanding of the role that inflammation plays in AIS; (4) the fibrinogen level and NLR are recognized as markers of inflammation in the peripheral circulation; they are not specific neuroinflammatory markers and can be influenced by various factors, thus making it challenging to draw conclusions about their specific impact on certain neuroinflammatory pathways; and (5) despite our study provided some evidence that higher fibrinogen and neutrophil-to-lymphocyte ratio may be a predictor for poor response to intravenous thrombolysis among AIS patients, no treatment strategies were changed accordingly. To explore additional appropriate treatment among AIS patients with higher fibrinogen and neutrophil-to-lymphocyte ratio is what we will study in the near future.

## Conclusion

In conclusion, our research indicates that the higher fibrinogen level and NLR are associated with a poor early response to thrombolysis. Furthermore, combining the fibrinogen level and NLR may better predict the response to intravenous thrombolysis in patients with AIS. However, future studies are necessary to extend the results and to explore corresponding treatment strategies.

## Data availability statement

The raw data supporting the conclusions of this article will be made available by the authors, without undue reservation.

## Ethics statement

The studies involving humans were approved by Ethics Committee of the Affiliated Changsha Central Hospital, Hengyang Medical School, University of South China. The studies were conducted in accordance with the local legislation and institutional requirements. The human samples used in this study were acquired from primarily isolated as part of our previous study for which ethical approval was obtained. Written informed consent for participation was not required from the participants or the participants' legal guardians/next of kin in accordance with the national legislation and institutional requirements.

## Author contributions

MD: Data curation, Formal analysis, Writing – original draft, Writing – review & editing. KS: Data curation, Investigation, Writing – original draft, Writing – review & editing. YT: Data curation, Funding acquisition, Investigation, Writing – review & editing. SC: Conceptualization, Investigation, Software, Writing – review & editing. WX: Data curation, Methodology, Supervision, Writing – review & editing. GH: Investigation, Software, Visualization, Writing – review & editing. JH: Data curation, Methodology, Supervision, Writing – review & editing. HX: Project administration, Validation, Writing – review & editing. CW: Conceptualization, Data curation, Investigation, Methodology, Writing – review & editing. ZW: Conceptualization, Data curation, Methodology, Software, Supervision, Visualization, Writing – review & editing. FL: Data curation, Formal analysis, Methodology, Project administration, Software, Supervision, Validation, Writing – review & editing.
